# Extraction of Bioactive Compounds from Spent Coffee Grounds Using Ethanol and Acetone Aqueous Solutions

**DOI:** 10.3390/foods12244400

**Published:** 2023-12-07

**Authors:** Ibtissam Bouhzam, Rosa Cantero, María Margallo, Rubén Aldaco, Alba Bala, Pere Fullana-i-Palmer, Rita Puig

**Affiliations:** 1 Department of Industrial and building Engineering, University of Lleida (UdL), Pla de la Massa, 8, 08700 Igualada, Spain; ibtissam.bouhzam@udl.cat (I.B.); rosa.cantero@udl.cat (R.C.); 2 Department of Chemical and Biomolecular Engineering, University of Cantabria, Av. de Los Castros s/n, 39005 Santander, Spain; maria.margallo@unican.es (M.M.); ruben.aldaco@unican.es (R.A.); 3 UNESCO Chair in Life Cycle and Climate Change ESCI-UPF, Pg. Pujades 1, 08003 Barcelona, Spain; alba.bala@esci.upf.edu (A.B.); pere.fullana@esci.upf.edu (P.F.-i.-P.)

**Keywords:** spent coffee ground, extraction, chlorogenic acid, total polyphenols, caffeine, storage duration, storage conditions, kinetic study

## Abstract

Given global coffee consumption, substantial quantities of spent coffee grounds (SCGs) are generated annually as a by-product of brewing coffee. SCG, although rich in bioactive compounds, is nowadays disposed of. The objective of this study is to compare, for the first time and from the same SCG, the efficiency of ethanol–water mixtures and acetone–water mixtures for the recovery of total polyphenols, chlorogenic acid, and caffeine. Acetone at 20% (m/m) was the most convenient solvent to extract all three bioactive compounds simultaneously, yielding 4.37 mg of GAE/g SCG for total polyphenols, chlorogenic acid (0.832 mg 5-CQA/g SCG), and caffeine (1.47 mg/g SCG). Additionally, this study aims to address some challenges associated with the industrial-scale utilization of SCG as a raw material, encompassing factors such as pre-treatment conditions (natural drying and oven drying), storage duration, and the kinetics of the extraction process. No significant difference was observed between the natural drying and oven drying of SCG. In terms of storage duration, it is advisable to process the SCG within less than 3–4 months of storage time. A significant decline of 82% and 70% in chlorogenic acid (5-CQA) and caffeine contents, respectively, was observed after eight months of storage. Furthermore, the kinetic study for the recovery of total polyphenols revealed that the optimal extraction times were 10 min for acetone at 20% and 40 min for water, with a yield increase of 28% and 34%, respectively. What is remarkable from the present study is the approach considered, using the simplest operating conditions (minimal time and solvent-to-solid ratio, and ambient temperature); hence, at an industrial scale, energy and resource consumption and equipment dimensions can be together reduced, leading to a more industrially sustainable extraction process.

## 1. Introduction

The loss of biodiversity and unsustainable food waste, driven by a linear growth model, present interconnected global challenges with economic, environmental, and social repercussions amid increasing food demand [1,2,3,4].

Therefore, changing our food system and moving towards a more circular economy system is imperative to ensure resource efficiency and waste reduction, rebuild biodiversity, and address climate change issues [2]. In a circular economy, food systems need to be designed to cycle through the economy, where food loss and waste can be converted into a valuable resource [4]. Thus, research is needed to convert food waste into valuable resources.

Instant coffee manufacturing generates significant amounts of waste [5]. Typically, only about 30% of the mass of coffee beans can be extracted into the coffee that we drink, while the remaining 70% ends up as spent coffee grounds (SCG) with no commercial value. The high amount of waste generated highlights the need to develop innovative uses for spent coffee grounds [6,7,8].

Recent studies have revealed that spent coffee grounds still contain many biological components, such as polyphenols and caffeine, which exhibit important bioactive properties, including antioxidant and anti-inflammatory activities [5].

Plant polyphenols have attracted interest due to their potent antioxidant properties and their ability to protect against cancer development. Plant polyphenols include phenolic acids, flavonoids, and tannins, as well as the less prevalent forms of stilbenes and lignans. Phenolic acids can be divided into the following two classes: derivatives of benzoic acid, such as gallic acid, and derivatives of cinnamic acid, such as coumaric, caffeic, and ferulic acid [9]. The main phenolic acids reported to be present in spent coffee grounds are chlorogenic acids, and they belong to the second class (cinnamic derivatives) [10]. Chlorogenic acids (CGAs) belong to a group of esters that share structural resemblance with quinic acid (QA) and contain one or more cinnamate derivatives such as caffeic, ferulic, and p-coumaric acids [11]. The main groups of CGA are as follows: caffeoylquinic acids (CQA), with three isomers (3-, 4- and 5-CQA); dicaffeoylquinic acids (diCQA), with three isomers (3,4-diCQA; 3,5-diCQA; 4,5-diCQA); feruloylquinic acids (FQA), with three isomers (3-, 4- and 5-FQA); p-coumaroylquinic acids (pCoQA), with three isomers (3-, 4- and 5-pCoQA), and six mixed diesters of caffeoylferuloyl-quinic acids (CFAQ) [12]. Caffeoylquinic acids are the most abundant isomers found in SCG, which are thought to confer an array of health-enhancing advantages [13].

Caffeine is a widely used substance that is consumed in various forms, including coffee, tea, energy drinks, and dietary supplements. The primary source of caffeine is decaffeination. Spent coffee grounds can also be a viable source of caffeine extraction. Extracted caffeine is chemically identical to synthetic caffeine [14]. Therefore, the caffeine extracted from SCG can provide economic and environmental benefits and could be a promising alternative to synthetic caffeine.

Different methods have been developed for the extraction of polyphenols and caffeine from spent coffee grounds, including solid–liquid extraction, ultrasound extraction, microwave-assisted extraction, Soxhlet extraction, hydrothermal treatment, and other methods [5]. Among these techniques, solid–liquid extraction is the most widely used, requiring no specialized equipment [5,15]. Indeed, from the perspective of the sustainable valorization of SCG, avoiding toxic solvents is advised. Some authors recommended the use of polar solvents for the extraction of polyphenols, such as ethanol, methanol, or acetone [13]. On the other hand, caffeine is soluble in acetone, water, methanol, and ethanol [5]. Thus, ethanol and acetone could be used for the extraction of both polyphenols and caffeine.

Water is considered the greenest solvent overall; it has low toxicity, is nonflammable, non-explosive, naturally abundant, inexpensive, and can be readily separated from the reaction mixture [16]. Nevertheless, water alone does not always yield the desired extraction efficiency. On the other hand, some researchers have suggested that the addition of water to organic solvents, such as acetone, methanol, and ethanol, creates a more polar environment that facilitates the extraction of phenolic compounds [17]. Hence, ethanol–water and acetone–water mixtures can be used for the extraction of polyphenols.

Ethanol is a commonly used solvent because it has low toxicity, a low boiling point, and is readily available. These important characteristics make it an environmentally friendly alternative [18]. Furthermore, in the context of large-scale applications, ethanol emerges as a viable bio-solvent that can be generated from the process of alcoholic fermentation utilizing diverse sugar- or starch-containing feedstocks. Notably, the recycling potential inherent to ethanol augments its appeal as an environmentally sustainable option for process development [13].

Ethanol–water mixtures are widely used for the extraction of caffeine and total polyphenols. However, the influence of different proportions of ethanol–water on the extraction yield of total polyphenols, caffeine, and chlorogenic acid (all together) from the same SCG has scarcely been studied.

Additionally, despite numerous studies on the extraction of polyphenols, chlorogenic acid, and caffeine, the use of acetone as a solvent for their recovery has not yet been explored, even though it is considered a preferred solvent according to the solvent selection table of the Pfizer Company [19]. It is accessible, cost-effective, has a low boiling point, and completely dissolves in water.

On the other hand, it is challenging to find studies in the literature comparing various solvents for the simultaneous extraction of total polyphenols, chlorogenic acid, and caffeine from spent coffee grounds (SCG). Many papers tend to focus on introducing novel extraction procedures or optimizing conditions for already established methods. However, if the objective is to efficiently reuse food waste, such as SCG, into a valuable resource that is rich in health-enhancing compounds, it is imperative to compare diverse extraction alternatives using the same SCG, as previously justified by Bouhzam et al. [5].

Therefore, the objective of the present paper is to compare the efficiency of ethanol–water mixtures and acetone–water mixtures for the recovery of total polyphenols, chlorogenic acid, and caffeine from the same SCG. A global aim is to contribute to the circularity of food waste through a practical example. Experiments were performed under the simplest conditions described in the literature, including room temperature, the lowest liquid-to-solid ratio (5.7 mL/g SCG), and a short extraction time (1 min) simultaneously [20]. This study provides new insights into the influence of different solvent mixtures on the extraction yield and explores, for the first time, the potential of acetone–water mixtures as an alternative solvent for the extraction. Additionally, this study aims to address some challenges associated with the industrial-scale utilization of SCG as a raw material, encompassing factors such as storage pre-treatment and duration and the kinetics of the extraction process.

## 2. Materials and Methods

### 2.1. Spent Coffee Grounds (SCG)

Wet spent coffee grounds, derived from mixtures of Arabica and Robusta coffee varieties of the brand “Novell gourmet”, were supplied by a coffee bar in the province of Barcelona (Spain). The mixture was dried naturally for one week and subsequently stored at room temperature in plastic capsules, covered but not hermetically sealed, for future extractions. Spent coffee grounds were originally obtained from infusions of 100% caffeinated coffee beans.

In Section 3.1.1, where the influence of the drying method was investigated, the SCG used was a blend resulting from the preparation of caffeinated (80% approximately) and decaffeinated (20%) coffee infusions. SCG was collected and separated into two fractions; one was left to dry naturally for 7 days (temperature: 23 °C, and humidity: 50%), while the other was dried in an oven at 50 °C for 24 h. Then, both fractions were stored in plastic capsules at room temperature, covered but not sealed hermetically.

### 2.2. Chemicals and Reagents

Acetonitrile (99.9%), ethanol (99.9%), acetone (99.8%), glacial acetic acid (99.8%), and Folin–Ciocalteus’ phenol reagent were supplied from Scharlab (Barcelona, Spain). Sodium carbonate was purchased from Acros Organics (Geel, Belgium).

The standards used were as follows: 5-chlorogenic acid (5-O-Caffeoylquinic acid, 5-CQA, 98%) caffeine (1,3,7-trimethylxantine), and the HPLC grade were purchased from Sigma-Aldrich (St. Louis, MO, USA). Gallic acid (98%) was supplied from Scharlab (Barcelona, Spain). The chemicals were all of the analytical reagent grade.

A Milli-Q System, equipped with a 0.22-µm filter from Merck Millipore (Burlington, MA, USA), was used to prepare ultrapure water.

### 2.3. Equipment

The quantification of caffeine (Caf) and chlorogenic acid (5-CQA) was performed using a Waters 2695 high-performance liquid chromatography (HPLC) coupled to a 2998 UV Detector (Milford, MA, USA). A reverse phase column C8 (5 µm particle size, 100 Å pore size, 150 mm length, and 4.6 mm internal diameter) was provided by Scharlab (Barcelona, Spain). The data were processed using the Empower Solutions 2.0 Software (Orlando, FL, USA).

The quantification of the total polyphenols content was conducted using a Perkin Elmer 501S09110511 UV-spectrophotometer (Barcelona, Spain).

A J.P. Selecta ES-05 oven was used to dry SCG (Barcelona, Spain). For sample preparation, a Velp Scientifica F202A0176 vortex shaker (Usmate, MB, Italy) was used together with a J.P. Selecta 7002575 centrifuge and a 3000865 ultrasound bath (Barcelona, Spain).

### 2.4. Solid/Liquid Extraction

The efficiency of ethanol and acetone–water mixtures for total polyphenols, chlorogenic acid (5-CQA), and caffeine (Caf) extraction was investigated under the simplest conditions (room temperature, 5.7 mL/g SCG solvent/solid ratio, and 1 min extraction time). Different ratios of ethanol/water mixtures (0, 20, 40, 60, 80, and 96%) and acetone/water mixtures (0, 20, and 40%) were tested. A 0.7 g sample of spent coffee grounds (SCG) was mixed with 4 mL of the studied solution in a tube and stirred in a vortex shaker at 3000 rpm for 1 min. The mixture was then centrifuged for 30 min at 4200 rpm and filtered with a syringe filter (0.45 μm) before HPLC analysis. For caffeine quantification, 1:3 dilutions were used for the extracts obtained with 60, 80, and 96% ethanol/water mixtures to better integrate the caffeine peak. All experiments were performed in duplicate.

In Section 3.1.1, the two drying methods (oven and naturally drying) were compared by subjecting the samples to vortex extraction using water or a 40% ethanol/water mixture as the solvents. The concentration of the two analytes in the resulting extracts was measured using high-performance liquid chromatography-ultraviolet (HPLC-UV).

### 2.5. Analysis of Caffeine and Chlorogenic Acid by HPLC-UV

**Figure 1:**
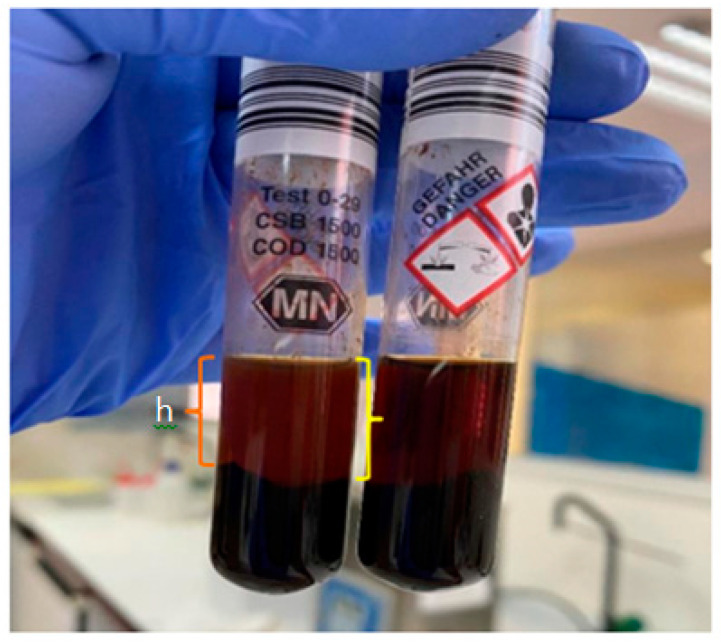
Extraction with acetone at 20% (m/m) (**right**) and 40% (m/m) (**left**): extract (**upper phase**) and SCG (**lower phase**).

HPLC-UV analysis was performed using a mobile phase consisting of 92% *v*/*v* water and 8% *v*/*v* acetonitrile, which were both acidified with 0.1% (*v*/*v*) acetic acid in the isocratic mode. The flow rate was 0.6 mL/min for 20 min. The sample injection volume was 20 µL at 36 °C, and the wavelength was set at 278 nm. Chlorogenic acid and caffeine calibration curves were used to quantify both substances in the samples. Both standards were prepared in ultrapure water in the concentration range of 40.4–404 mg/L for chlorogenic acid and 121.2–606 mg/L for caffeine. Chlorogenic acid has different isomers, which can be measured as follows: 3-CQA, 4-CQA, and 5-CQA. In the present study, the standard used was 5-O-caffeoylquinic (5-CQA). Thus, the results show the yield of only this isomer, 5-CQA. The results for both analytes, caffeine, and chlorogenic acid, were the average of two replicates and were expressed as concentrations in mg/L of the extract (ppm) and as the mg of the analyte per g dry SCG (including their coefficients of variation). The results in milligram analyte/g dry SCG were obtained by multiplying the concentration (ppm) with the volume of the extract obtained. The volume of the extract in the test tube was calculated using the cylindrical volume formula (V = πr^2^h), and the height was measured using a digital caliper. Therefore, it is important to note that the results expressed as mg/g SCG carry a higher uncertainty owing to the volume measurement (see Figure 1).

### 2.6. Analysis of Total Polyphenols with Folin-Ciocalteau Method

Total polyphenols content (TPC) was determined using the Folin–Ciocalteu method adapted from other studies described in the literature [21,22,23]. Following a series of preliminary experiments, several key parameters were identified as critical for the process. These include maintaining the pH of the solution within the range of 9–10 after the addition of the Folin reagent 2N, employing a sodium carbonate concentration of 20% m/m, maintaining a specific ratio of Folin to sodium carbonate at 1:3, and allowing for a color development time of 2 h in darkness before conducting UV testing. An aliquot of 0.1 mL of the extract (previously diluted with water 1:4 *v*/*v*) was mixed with a 0.5 mL Folin–Ciocalteu reagent and 1.5 mL of sodium carbonate (20% m/m), all diluted to 10 mL with ultrapure water. The mixture was then incubated in the dark at room temperature for 2 h for color development. Absorbance was then measured at 765 nm. Gallic acid calibration curves were obtained within a concentration range of 50–600 ppm.

The results were the average of four experiments and were reported as concentrations in ppm and as milligrams of gallic acid equivalents per gram of the dried SCG (mg GAE/g), including their coefficients of variation. As mentioned previously, the results expressed as mg/g SCG have higher uncertainty owing to the measurement of the volume.

### 2.7. Effect of Extraction Time on Polyphenols Content

Several experiments were performed under the same conditions but with different extraction times. A sample of 0.7 g of dry SCG was mixed with 4 mL of water or a mixture of acetone and water (20% m/m). Water and acetone 20% were chosen as solvents for the kinetic experiments: the first as a reference and the second because it was the one giving the best results for the three analytes simultaneously (polyphenols, caffeine, and chlorogenic acid) (see the Results and Discussion section).

The mixture was then stirred using a vortex shaker for the following different times: 1, 10, 20, 40, and 60 min for water extraction and 1, 10, 20, and 40 min for acetone 20% extraction. Subsequently, the mixture was centrifuged for 30 min at 4200 rpm, and the obtained extract was used for the determination of polyphenols. The results are the average of four experiments and are reported as concentrations in ppm and milligrams of gallic acid equivalents per gram of dried SCG (mg GAE/g SCG), including their coefficients of variation.

## 3. Experimental Results and Discussion

### 3.1. Storage of Spent Coffee Ground (SCG) after Drying

#### 3.1.1. Influence of Drying Method

The present study investigated the effects of two different drying methods, including natural drying (at room temperature for 7 days) and oven drying (at 50 °C for 24 h), on the contents of chlorogenic acid (5-CQA) and caffeine in spent coffee grounds.

In these experiments, the SCG used was a blend resulting from the preparation of caffeinated (80% approximately) and decaffeinated (20%) coffee infusions.

**Figure 2:**
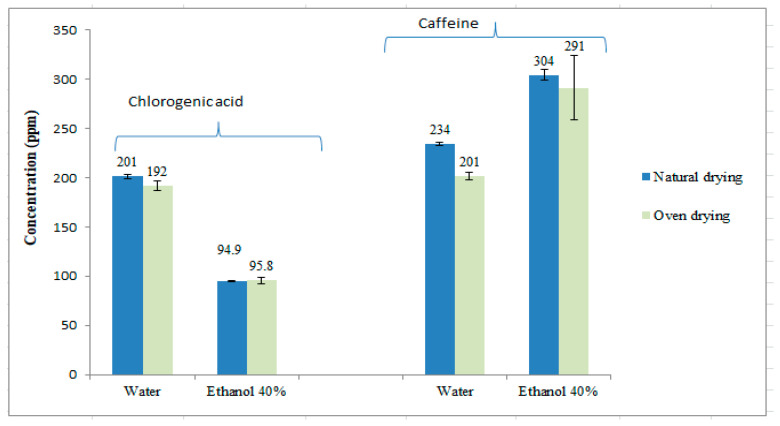
Comparison between natural drying (7 days at room temperature) and oven drying (24 h at 50 °C) SCG for the extraction of chlorogenic acid (5-CQA) and caffeine. The results are the average of duplicate experiments and are presented with their standard deviation.

As shown in Figure 2, no significant differences (they are within their variation coefficients) were observed in the concentrations of chlorogenic acid and caffeine between the two drying methods for either extraction solvent. Therefore, natural drying or oven drying can be used as viable methods for drying SCG without affecting the concentration of chlorogenic acid and caffeine. Further continued studies presented in this paper were performed using natural dried SCG.

Figure 2 also illustrates that ethanol 40% is a more efficient solvent than water for extracting caffeine, whereas the opposite is observed in the case of chlorogenic acid. This distinction arises from the varying polarity and chemical characteristics of the two bioactive molecules. Chlorogenic acid possesses an acidic group and five hydroxyls that are capable of forming hydrogen bonds with water, whereas caffeine, with two less polar carbonyl groups, exhibits a preference for a solvent with a higher organic content, such as ethanol at 40%.

#### 3.1.2. Influence of Storage Time

The influence of SCG storage time on the extraction of chlorogenic acid (5-CQA) and caffeine was studied over a period of eight months. The samples were analyzed at the following four time points: initially (time 0), after one month, four months, six months, and eight months of storage. Vortex extraction using water was employed to extract both compounds, which were analyzed through HPLC-UV.

**Figure 3:**
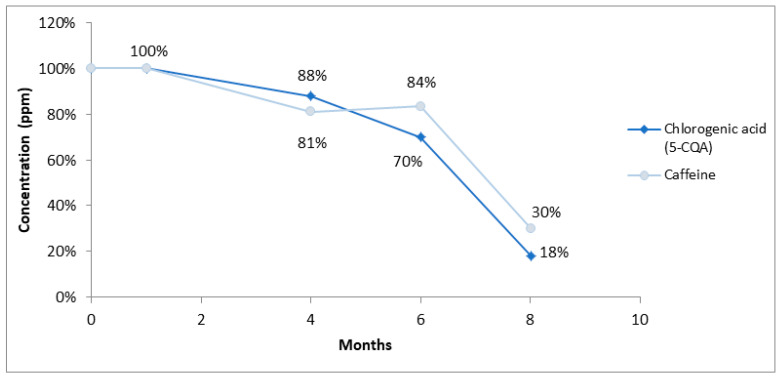
Evolution of chlorogenic acid (5-CQA) and caffeine concentrations in SCG from zero to eight months of storage.

The results presented in Figure 3 reveal a substantial decrease in the concentrations of chlorogenic acid (5-CQA) and caffeine by 82% and 70%, respectively, after an eight-month storage period.

This study suggests that the prolonged storage of SCG may lead to a significant reduction in the concentration of bioactive compounds. Hence, it is recommended to limit the storage time to less than 4 months to maintain initial levels of caffeine and chlorogenic acid as close as possible to the initial ones.

### 3.2. Extraction of Caffeine and Chlorogenic Acid Using Ethanol/Water Mixtures

**Figure 4:**
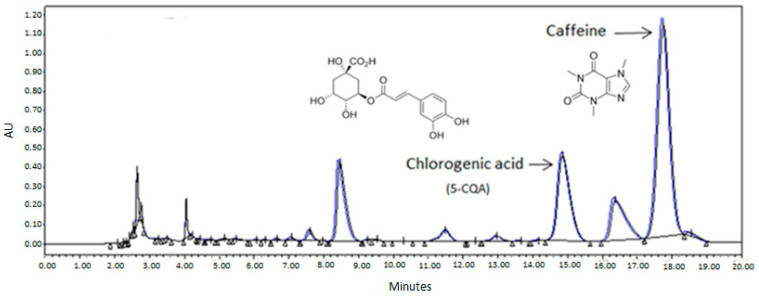
HPLC chromatogram of duplicate solid–liquid extractions using water.

The effects of hydroalcoholic solvents with different EtOH/water ratios (0, 20, 40, 60, 80, or 96% m/m) were investigated for chlorogenic acid and caffeine extraction. The chromatographic analysis of the obtained extract (shown in Figure 4) revealed the presence of distinct chlorogenic acid isomers. Spiking experiments were performed using a chlorogenic acid standard (5-CQA, 98%) at a concentration of 400 ppm to identify the peaks corresponding to the standard. The peak area marked in Figure 4 was the one that increased by a factor of 2.53 when the sample was spiked with 4 mL of the chlorogenic acid standard (400 ppm). Therefore, this peak was used for chlorogenic acid (5-CQA) quantification.

**Figure 5:**
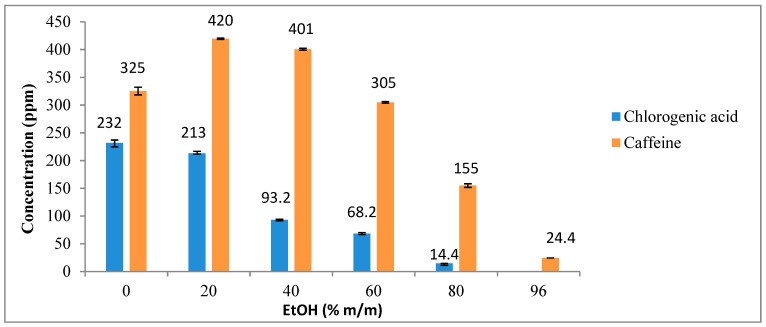
Influence of EtOH/water ratio on chlorogenic acid (5-CQA) and caffeine extraction. The results are the average of duplicate experiments and are presented with their standard deviation. Results in ppm.

The best result for chlorogenic acid (232 ppm) was observed with water extraction (or 0% ethanol, Figure 5), whereas chlorogenic acid was not detected in the extractions using 96% ethanol as the solvent. It should be noted that the yield of chlorogenic acid decreased progressively as the concentration of ethanol in the solvent mixture increased. The highest caffeine content was obtained with 20–40% ethanol (420–401 ppm, respectively, and, thus, a 5% variation). The caffeine concentration in the extract increased from 325 to 420 ppm, between 0% and 20% ethanol, and then decreased to 24.4 ppm with 96% ethanol (Figure 5). This can be justified by the duality of caffeine, combining both polar and non-polar groups within the same molecule. Initially, the addition of ethanol to water amplifies the organic characteristics of the solvent. Yet, beyond a specific ethanol concentration, the hydrogen bonds between caffeine and water diminished, subsequently decreasing its solubility in the solution.

**Figure 6:**
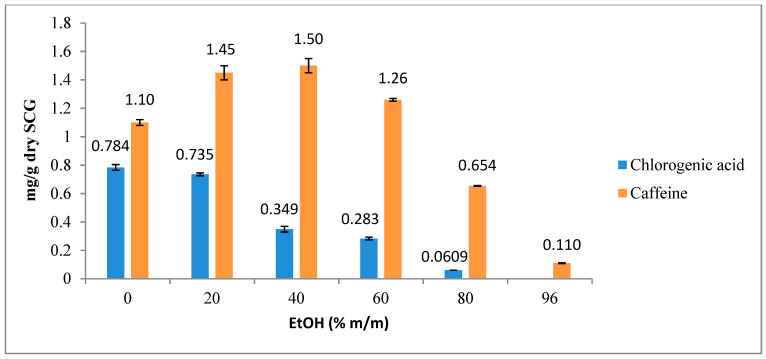
Influence of EtOH/water ratio on chlorogenic acid (5-CQA) and caffeine extraction. The results are the average of duplicate experiments and are presented with their standard deviation. Results in mg/g of dry SCG.

Figure 6 just shows the same results but expressed in mg/g of SCG instead of the concentration in the extract solution.

Hence, water is a better solvent to extract chlorogenic acid, while a 20–40% ethanol solvent is preferable to obtain caffeine. This can be explained, as said before, by the nature of chlorogenic acid and caffeine (Figure 4 shows the chemical structures of both molecules). Chlorogenic acid has five hydroxyl (OH) groups and one acidic group, enabling its solubility in water and facilitating the formation of hydrogen bonds with water molecules. On the other hand, caffeine lacks hydroxyl groups and predominantly contains two carbonyl groups, which have lower polarity compared to the hydroxyl and acidic groups. As a result, while chlorogenic acid is more soluble in water, caffeine demonstrates a higher affinity for mixtures of water with an organic solvent at a specific concentration, making a 20–40% ethanol solvent preferable for its extraction [14,15].

### 3.3. Extractions Using Acetone/Water Mixtures

The influence of acetone/water mixtures (with 0, 20, and 40% acetone ratios) on chlorogenic acid and caffeine extraction was evaluated.

As mentioned in the previous section, spiking experiments were performed with a chlorogenic acid standard (5-CQA, 98%) to determine the peak corresponding to the standard.

**Figure 7:**
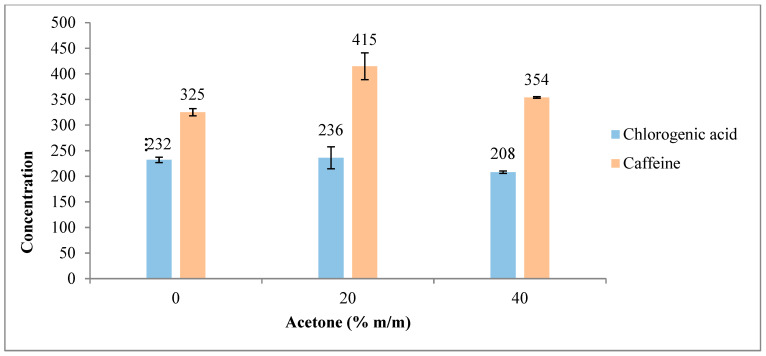
Influence of acetone/water ratio on chlorogenic acid (5-CQA) and caffeine extraction. The results are the average of duplicate experiments and are presented with their standard deviation. Results in ppm.

**Figure 8:**
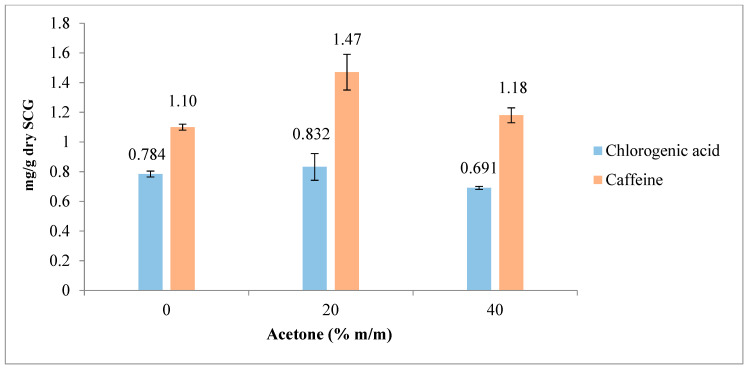
Influence of acetone/water ratio on chlorogenic acid (5-CQA) and caffeine extraction. The results are the average of duplicate experiments and are presented with their standard deviation. Results in mg/g of dry SCG.

The yields of chlorogenic acid (5-CQA) and caffeine obtained using different acetone/water ratios are shown in Figure 7 and Figure 8, expressed in ppm and mg/g SCG, respectively. As illustrated in the figures, the best result for caffeine extraction was achieved using 20% acetone. However, for chlorogenic acid (5-CQA), the extraction results were comparable between water and 20% acetone solvents. An increase in the acetone concentration beyond 20% resulted in a decrease in both chlorogenic acid and caffeine yields. This can be attributed to a reduction in solubility due to the diminishing of hydrogen bonds that both bioactive compounds can form with water.

### 3.4. Total Polyphenols Content

**Figure 9:**
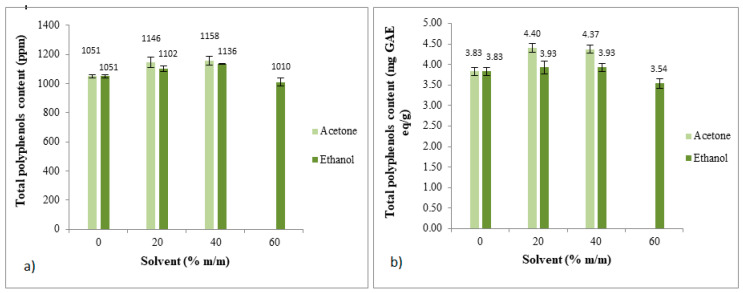
Total polyphenols content (TPC) as a function of different acetone/water and ethanol/water ratios. The results are the average of quadruplicate experiments and are presented with their standard deviation. (**a**) Results in ppm. (**b**) Results in mg GAE eq/g of dry SCG.

Water and water mixtures with ethanol (20%, 40%, and 60%) or acetone (20% and 40%) were used as solvents to extract polyphenols from spent coffee grounds (SCG) (Figure 9). The total polyphenols content (TPC) in the extract was measured in terms of the concentration (ppm) or as milligrams of gallic acid equivalents per gram of SCG (mg GAE eq/g SCG). The results (Figure 9) reveal that there were no significant differences in the results between the use of 20% or 40% (m/m) acetone or ethanol, while ethanol at 60% (m/m) resulted in a decrease in total polyphenols. Consequently, to minimize the volume of organic solvent, 20% (m/m) acetone or ethanol is recommended to conduct the experiments.

### 3.5. Comparison of Solvents to Extract Total Polyphenols, Chlorogenic Acid (5-CQA), and Caffeine

**Figure 10:**
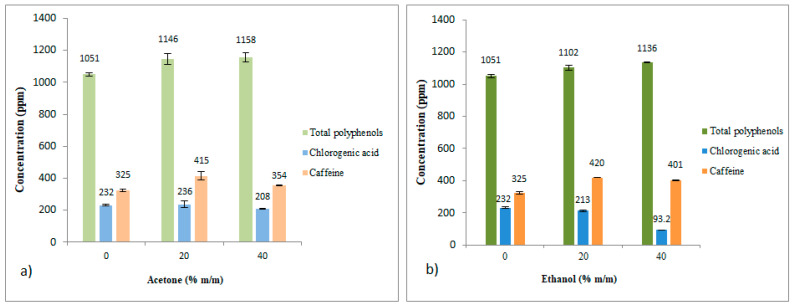
Total polyphenols, chlorogenic acid (5-CQA) and caffeine contents as a function of different acetone/water and ethanol/water ratios. (**a**) Results for acetone–water mixtures (ppm). (**b**) Results for ethanol–water mixtures (ppm).

**Figure 11:**
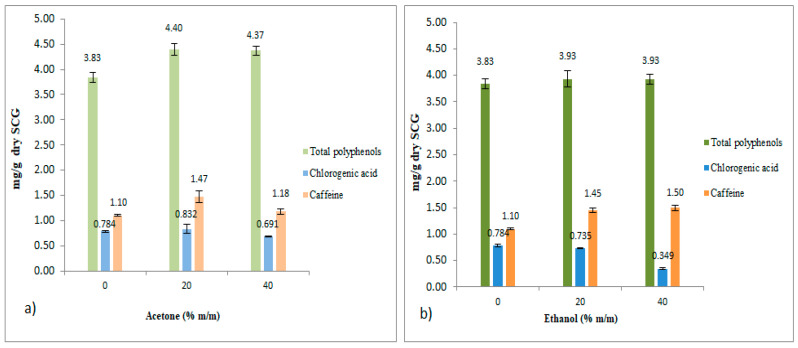
Total polyphenols, chlorogenic acid (5-CQA) and caffeine contents as a function of acetone/water and ethanol/water ratios. (**a**) Results for acetone–water mixtures (mg/g dry SCG). (**b**) Results for ethanol–water mixtures (mg/g dry SCG).

Results from previous sections are included together in Figure 10 and Figure 11 for comparison purposes.

The results revealed that acetone (20% m/m) and ethanol (20% m/m) were more effective than water in extracting phenolic compounds and caffeine (9% and 28% higher yield, respectively). However, in the case of chlorogenic acid (5-CQA), water and acetone (20% m/m) were slightly better (about 11%) than ethanol (20% m/m).

### 3.6. Effect of Extraction Time at Room Temperature

**Figure 12:**
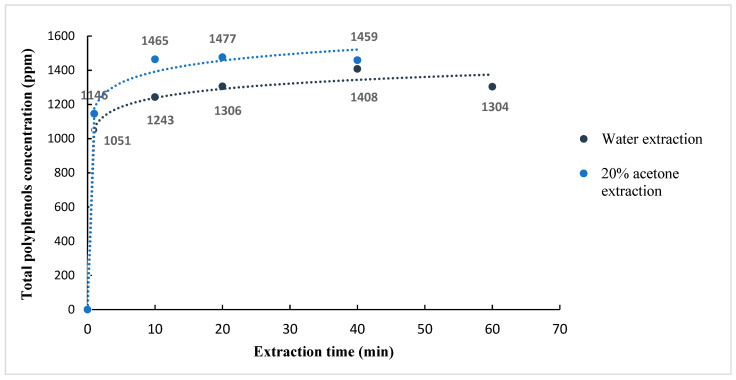
Kinetic curves of total polyphenols at room temperature using water and acetone at 20% (m/m) as the extraction solvents. The results are the average of quadruplicate experiments.

Increasing the extraction time has been identified as a potential factor for enhancing extraction efficiency, as previously mentioned [5]. In this kinetic study, separate applications of acetone (20% m/m) and water were employed as extraction solvents at room temperature. Figure 12 illustrates the influence of varying extraction times on the total polyphenols content in the SCG extracts.

The fastest extraction rate occurred between 0 and 1 min. Beyond this time, the slope of the curve diminished. Hence, after 1 min, a significant portion of the total polyphenols had already been extracted, leading to a subsequent slowdown in the extraction process.

Nevertheless, in the case of water, increasing the extraction time from 1 to 40 min, the total polyphenols content of the extracts increased by +34% (from 1051 to 1408 ppm). For acetone at 20% (m/m), an increase in the total polyphenols content (+28%) was observed over the extraction time range (0–10 min). Thus, the maximal yield of polyphenols was reached faster with Acetone at 20% (within 10 min) than with water (within 40 min). However, in the case of water extraction, increasing the extraction time from 10 to 40 min resulted in an increase of only 13%. This means that 10 min might be considered a sufficient extraction time for both solvents.

**Figure 13:**
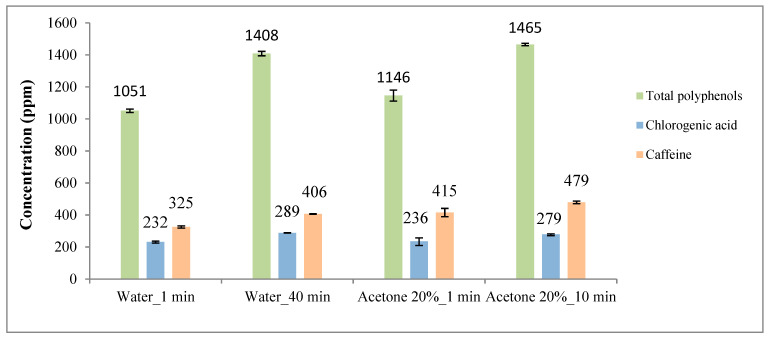
Chlorogenic acid (5-CQA) and caffeine contents in water and acetone at 20% (m/m) at different extraction times. The results for total polyphenols are the average of quadruplicate experiments, and for chlorogenic acid and caffeine, are the average of duplicate experiments presented with their standard deviation. Results in ppm.

**Figure 14:**
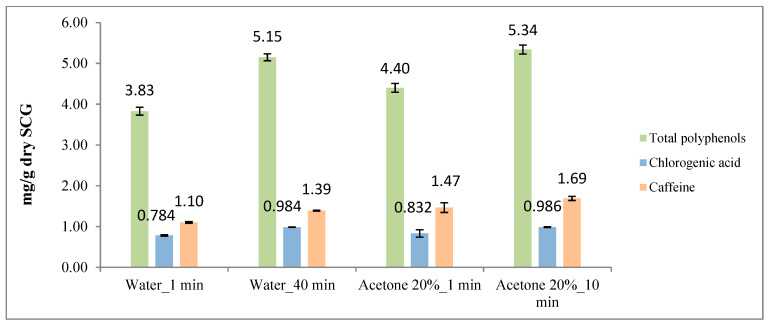
Chlorogenic acid (5-CQA) and caffeine contents in water and acetone at 20% (m/m) and different extraction times. The results for total polyphenols are the average of quadruplicate experiments, and for chlorogenic acid and caffeine are the average of duplicate experiments and are presented with their standard deviation. Results in mg/g.

Chlorogenic acid (5-CQA) and caffeine yields were also determined at the optimal extraction times for water and acetone at 20% (40 and 10 min, respectively). These results were obtained using HPLC-UV and are presented along with the total polyphenol yields in Figure 13 and Figure 14. These were the best solvents and extraction times identified in this study.

The results indicate a significant influence of the extraction time and solvent type on the content of these bioactive compounds. Extending the extraction time (from 1 to 40 min for water) resulted in a reasonable yield increase for both chlorogenic acid and caffeine (25% and 25%, respectively). Similarly, using acetone at 20% as the solvent resulted in an 18% increase in chlorogenic acid yield and a 15% increase in caffeine yield when the extraction time was extended from 1 to 10 min.

The results show a more significant increase in the total polyphenols yield over time compared to the increases observed in chlorogenic acid and caffeine (Figure 13), which can be attributed to the comprehensive nature of total polyphenols, including a great diversity of compounds quantified collectively using the Folin method and expressed as mg GAE eq/L. Consequently, these compounds may not present the same behavior in the extraction process as caffeine and chlorogenic acid.

### 3.7. Critical Discussion of the Present Results Compared with the Literature

First of all, it is noteworthy to acknowledge that results from different studies are not directly comparable because they use SCG from different origins and with different compositions due to inherent variations resulting from the coffee variety, roasting degree, and coffee infusion preparation methodologies. These variables can exert considerable influence on the concentration of bioactive compounds within the original coffee matrix, thereby potentially affecting the extraction outcomes of SCG and limiting direct comparisons across studies, as stated by some authors [5,23,24,25].

Nevertheless, a literature review was performed to identify all the studies in the literature that have recovered total polyphenols, chlorogenic acid, or caffeine using solid–liquid extraction, and the same solvents were evaluated in this study. A total of 20 papers were identified (some of which are included in Table 1 for discussion purposes).

Several studies have used water and ethanol–water mixtures as extraction solvents. However, only one study employing ethanol 25% quantified all three types of analytes (polyphenols, caffeine, and chlorogenic acid) simultaneously, as conducted in the present study for different ethanol percentages. Among the identified studies, the majority (18 out of 20) analyzed the total polyphenol content. Some of them also quantified caffeine, while only two studies measured the chlorogenic acid content. However, these last two studies did not specify whether they measured only one isomer of chlorogenic acid or included all its isomers in the quantification. The recovery rates reported in the literature vary significantly, ranging from 3.9 to 33.07 mg GAE/g for total polyphenols, from 0.02 to 4.8 mg/g for chlorogenic acid, and from 0.15 to 11.50 mg/g for caffeine.

On the other hand, the use of acetone/water mixtures for extracting these bioactive substances from SCG has not been previously reported in the literature. It is worth mentioning that only one study by Da Silva Araújo et al. [26] used acetone to solely investigate the use of 100% acetone for total polyphenols (TPC) recovery (see Table 1, n° 4), further highlighting the novelty of the present study.

Table 1 presents the different solid liquid extraction studies found in the literature. Studies 1 to 3 show a comparison of TPC, 5-CQA, and the caffeine contents for different SCG types, highlighting that results from different SCGs are not comparable even using the same extraction conditions. On the other hand, studies 4 to 13 present the influence of ethanol–water proportions on the recovery of TPC or caffeine. Only the best ethanol proportion that yielded better results for TPC or caffeine is presented in the table. Lastly, study 14 illustrates the impact of the liquid-to-solid ratio and temperature variations on TPC.

In the present study, and for ethanol–water mixtures, the best results were obtained using ethanol at 20–40% for TPC and caffeine and ethanol at 0–20% for 5-CQA (see Figure 10 and Figure 11). These optimal ethanol–water proportions are in agreement with two other authors [14,26]. However, other studies reported better TPC results using ethanol at 50% [13,15,27,28] or ethanol at 60% [26,29,30,31,32]. Nevertheless, these studies did not measure 5-CQA, with most of them only investigating TPC. These variations in the optimal proportions of ethanol among the different studies highlight the complexity of obtaining a global optimization, which requires considering all the variables that affect the process, such as the type of coffee and its production process, as well as the preparation of the infusion and all the operating conditions for the extraction of bioactive compounds from SCG.

On the other hand, when comparing the extraction conditions (such as extraction time, temperature, and solvent-to-solid ratio) of this study with those reported in the literature, it is evident that the current study used the shortest extraction time (1 min), ambient temperature, and a minimal solvent-to-solid ratio (5.7 mL/g). These specific operating parameters are likely to exert a significant influence on the extraction yield. For example, as reported in [30], an increase in the liquid-to-solid ratio from 10 to 50 mL/g led to a considerable increase in the extraction (40%; from 12.12 to 16.94 mg GAE/g) (Table 1, n° 14) and elevating the temperature from 20 to 60 °C resulted in 38% yield augmentation (Table 1, n° 14).

Moreover, the extraction time is also an important factor in the extraction of bioactive compounds (as demonstrated in Section 3.6).

The present study was designed with the objective of working with a minimal solvent-to-solid ratio and ambient temperature. This choice was driven by our interest in obtaining highly concentrated analytes while minimizing the need for extensive solvent removal when using them to produce a new product (as the evaporation process consumes significant energy resources). Simultaneously, we aimed to minimize energy consumption associated with heating processes. The reduction in solvent use not only aligns with energy reduction but also with resource conservation by decreasing solvent use and subsequent industrial equipment dimensions to perform the process at an industrial scale. This approach can contribute to obtaining a more industrially sustainable process. Hence, it might be worth enhancing the present results by conducting the extraction at higher temperatures and a solvent/solid ratio if the gain in terms of yield or efficiency is significant enough to offset the investment in energy and the material resources required. Therefore, it is advisable to conduct further research to evaluate, considering both environmental and economic factors, whether the augmented yield justifies the subsequent increase in energy and resource consumption. This analysis should involve a multi-objective optimization approach.

**Table 1:** Examples of solid/liquid extraction methods reported in the literature.

Study n°	Year of Publication	Solvent	t (min)	T (°C)	(mL/g) Solvent-to-Solid Ratio	TPC (mg GAE/g)	Caffeine (mg/g)	Chlorogenic Acid (mg/g)	Reference
1	2019	Ethanol 25%	15	60	83	18 *	1.9 *	0.02 *	[23]
						26 *	6 *	4.8 *	
						9 *	0.3 *	0.8 *	
2	2011	Ethanol 70%	120	50	40	19.98 *	-	-	[24]
						17.09 *	-		
3	2013	Ethanol 60%	30	60	50	28.26 *	11.50 *	-	
						23.9 *	5.99 *		
		Water	30	60	50	19.62 *	11.23 *	-	[25]
						17.43 *	6.00 *		
4	2021	Ethanol 15%	90	60	30	8.6	-	-	
		Acetone 100%	90	65	30	3	-	-	[26]
5	2021	Ethanol 20%	15	Room temperature	25	-	4.1	-	[14]
6	2023	Ethanol 50%	30	50	40	9	-	-	[13]
7	2023	Ethanol 50%	69	65	28	17.79	4.37	-	[15]
8	2023	Ethanol 60%	90	60	40	33.07	-		[29]
9	2013	Ethanol 60%	105	40	30	14.06	-	-	[30]
10	2011	Ethanol 60%	60	25	15	11.50	-	-	[31]
11	2015	Ethanol 50%	90	60	30	23.79	-	-	[27]
12	2019	Ethanol 50%	24 h	4	10	8.4	-	-	[28]
13	2022	Ethanol 66%	-	60	50	22.02	-	-	[32]
14	2013	Ethanol 60%	180	60	10	12.12			[30]
			180	60	50	16.94	-	-	
			180	20	50	12.28			

* Results for different SCG types.

## 4. Conclusions

The present study aims to promote circularity in addressing popular food waste, especially spent coffee grounds, by extracting valuable bioactive compounds with different solvents (total polyphenols, chlorogenic acid, and caffeine) that present different applications in the pharmaceutical and food industries.

The paper investigates water–acetone mixtures as an alternative solvent for this extraction. Additionally, other aspects, such as storage conditions and the kinetics of the extraction process, have also been studied. SCG needs to be dried and stored for less than 3–4 months before extraction to prevent analyte degradation. The kinetics of the extraction at room temperature show that about 70% of polyphenols can be extracted within just 1 min, using either water or acetone at 20% as solvent.

Acetone at a concentration of 20% was the most convenient solvent for the extraction of total polyphenols (1146 ppm or 4.40 mg GAE/g SCG), chlorogenic acid (236 ppm or 0.832 mg 5-CQA/g SCG), and caffeine (415 ppm or 1.47 mg caf/g SCG) simultaneously. These results were obtained using the simplest conditions, including room temperature, the lowest liquid-to-solid ratio (5.7 mL/g SCG), and a short extraction time (1 min) simultaneously. Therefore, they can be enhanced by conducting the extraction at a higher temperature and solvent/solid ratio if the gain in terms of yield or efficiency is significant enough to offset the investment in energy and material resources required.

The present study was conceived with the aim of operating under the most straightforward conditions to achieve highly concentrated analytes in the shortest time and with the lowest energy consumption. This decision was driven by our commitment to reduce environmental and economic costs linked to energy and resource utilization. Decreasing solvent use not only enhances the energy efficiency of analyte concentration but also contributes significantly to resource conservation. By minimizing the use of solvents, there is a consequential reduction in the dimensions of industrial equipment needed for scaling up the process. These insights, often overlooked at the laboratory level, provide a basis for more sustainable industrial processes.

## Data Availability

Data are contained within the article.
